# Review of quinoa fermentation: product diversity, process optimization, and nutritional enhancement

**DOI:** 10.3389/fnut.2025.1605558

**Published:** 2025-09-16

**Authors:** Chen Li, Tong Liu, Xiaohong Li, Wenli Gao, Jiayin Lv, Gaojian Hu, Chunjiang Li, Fangfang Liu, Xianjun Liu, Xianglong Meng

**Affiliations:** ^1^College of Biological and Food Engineering, Jilin Engineering Normal University, Changchun, China; ^2^Department of Gastrointestinal Colorectal and Anal Surgery, The China-Japan Union Hospital of Jilin University, Changchun, China; ^3^Laboratory of Micro and Nano Biosensing Technology in Food Safety, Hunan Provincial Key Laboratory of Food Science and Biotechnology, College of Food Science and Technology, Hunan Agricultural University, Changsha, China; ^4^Department of Orthopedics, The China-Japan Union Hospital of Jilin University, Changchun, China; ^5^Jilin Province Hua En Biotechnology Co. Ltd., Changchun, Jilin, China; ^6^Department of Neurology, Jilin City Central Hospital, Jilin, China; ^7^Department of Gastroenterology and Hepatology, China-Japan Union Hospital, Jilin University, Changchun, China

**Keywords:** quinoa, fermentation process, nutritional value, beverages, dairy products, condiments

## Abstract

Fermented quinoa has emerged as a promising functional food owing to its enhanced nutritional profile, improved bioactive compound bioavailability, and favorable sensory attributes. Key fermentation parameters-microbial selection, process conditions, and substrate pretreatment-that govern the quality and functionality of fermented quinoa products. It highlights microbial-driven biotransformation of polyphenols and flavonoids, which enhances antioxidant activity and bioavailability. Fermentation also modulates sensory profiles and promotes gut health through enrichment of beneficial genera. These data provide a foundational framework for process standardization, scale-up, and industrial adaptation, particularly highlighting the versatility of lactic acid bacteria and the need for mechanized fermentation technologies to enhance commercial viability. Future research should focus on multi-omics approaches to decipher microbial consortia dynamics, *in vivo* validation of health benefits, development of clean-label formulations, and exploration of sustainable fermentation technologies. This review provides a scientific foundation for optimizing quinoa-based biotransformation processes and accelerating the development of next-generation fermented quinoa products with enhanced nutritional and health-promoting properties.

## 1 Introduction

Quinoa, a low-glycemic index (GI) crop native to the Andes Mountains, has been cultivated for over 5,000 years. Known as the “super grain,” “future food,” and “mother of food” (1), it is prized for its exceptional nutritional value. The Food and Agriculture Organization of the United Nations recognizes quinoa as the one of the most critical single crop that provides all essential amino acids, trace elements, and vitamins humans need (2). Its balanced amino acid composition and comprehensive nutrition have made it a key research focus (3).

Despite growing market demand for quinoa-fermented products, knowledge gaps remain regarding fermentation processes and key influencing factors. This study comprehensively reviews quinoa-fermented products developed globally, providing an overview of the current product landscape. It also analyzes fermentation processes of representative products to identify optimal parameters and highlights critical factors affecting fermentation, essential for quality control and product improvement.

By addressing these areas, this research aims to guide future development of quinoa-fermented products, enhancing their nutritional value, quality, and market competitiveness. The findings not only bridge existing knowledge gaps but also support advancements in the quinoa deep-processing industry.

## 2 Nutritional value and function of quinoa

**Figure 1:**
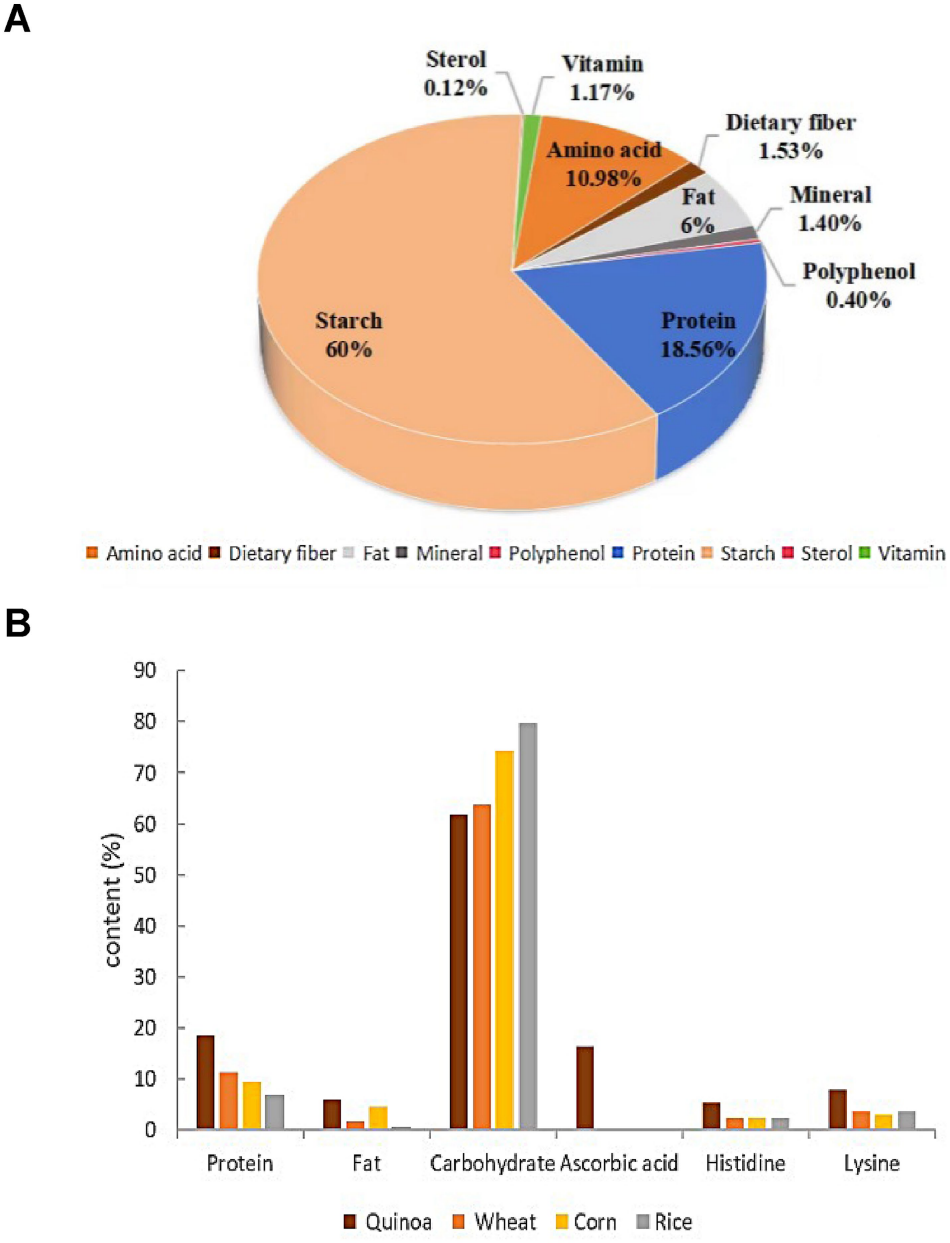
Chart of quinoa nutritional content. **(A)** Description of the specific nutrient composition in quinoa; **(B)** Comparing the content of major nutrients across different coarse grains.

Quinoa is a nutrient-rich crop, providing high-quality protein, essential amino acids, minerals, and omega-3 fatty acids. Its gluten-free nature makes it an ideal food for individuals with Crohn’s disease (CD) (4). As shown in Figure 1A, quinoa’s primary nutritional components are starch, protein, and amino acids. Its starch has a small particle size (0.4–2.0 μm) and high amylopectin content (5), giving it superior gelatinization and fermentation properties compared to other cereals. Quinoa contains 2.0–9.5% fat, 80% of which is unsaturated fatty acids, including squalene, which aids in fat reduction (6, 7). It also provides a well-balanced amino acid profile with over 17 types, making it the most nutritionally complete whole grain (8). Figure 1B shows that quinoa’s lysine content is double that of wheat and corn and 25% higher than rice, while its histidine levels match corn and exceed rice and wheat (9). Rich in minerals, vitamins, and soluble dietary fiber (1.53%), quinoa meets the nutritional needs of pregnant women in later stages, particularly for K, Fe, Zn, VE, and folic acid (10). It also contains at least 26 phenolic compounds, such as vanillic and ferulic acids (11), along with functional components like flavonoids, rutin, quercetin, and phytosterols (12). These compounds help combat fatigue, boost immunity, and support chronic disease management, positioning quinoa as a promising functional food.

Despite its benefits, quinoa’s coarse texture and bitter-tasting compounds-such as saponins and phytic acid-limit its appeal. Saponins, concentrated in the pericarp, contribute to its strong bitterness (13), with up to 87 different types identified (14) and levels in the outer layer reaching 13.39% (15). Processing is required to reduce saponin levels below 0.06% for improved taste (16). Flavonoids and polyphenols also contribute to bitterness and astringency (17), while antinutritional factors like phytates (7.92–8.93 g/kg) hinder mineral absorption (18, 19). Fermentation offers an effective solution by converting saponins into less bitter derivatives like polysaccharides (20), enhancing nutrient bioavailability. It also generates antioxidants that stabilize free radicals, prevent oxidation, and improve flavor (21). Beneficial fermentation microbes aid digestion and nutrient absorption, while fermented quinoa products exhibit antioxidant, anti-inflammatory, and gut health benefits (22). These advantages highlight fermentation’s potential to transform quinoa into a more palatable and nutritionally optimized food.

## 3 Quinoa-fermented beverages

### 3.1 Quinoa alcoholic beverages

Quinoa alcoholic beverages are brewed primarily from quinoa, yielding various fermented drinks such as beer, liquor, fruit wine, and yellow wine through different fermentation methods and ingredient combinations. During fermentation, microorganisms break down quinoa’s sugars into alcohol, organic acids, and aromatic compounds while preserving many of its natural nutrients. This enhances both the nutritional value and flavor profile of the beverages. Beyond retaining quinoa’s health benefits, these fermented drinks stimulate digestive enzyme secretion, boost appetite, and aid digestion (23). The fermentation process also develops unique aromatic qualities, significantly improving their sensory characteristics.

#### 3.1.1 Quinoa beer

Beer is the world’s most popular alcoholic beverage, but traditional beer contains gluten, making it unsuitable for individuals with CD (22). Quinoa-based gluten-free beer offers a smoother taste, rich foam, and distinctive aroma, setting it apart from conventional beers (24). Quinoa beer has been widely researched globally.

The first quinoa beer was developed in 2005 by Zweytick et al. using quinoa and *German malt yeast* (25). Prasad et al. (26) later created a non-alcoholic quinoa beer (<0.5% vol.) with antioxidant properties using wild quinoa and Burmese Pichia pastoris. Dezelak (27) produced quinoa beer with buckwheat, noting that quinoa beer contained higher amino acid levels (25% more than buckwheat beer) and abundant metal cations. *Saccharomyces pastorianus tum* 34/70 was used for fermentation, and according to pierce classification, it showed that different fermentation strains had selectivity for amino acid absorption of different raw materials, which directly affected the nutritional composition of beer. Bian et al. (28) found that fermentation temperature and yeast quantity significantly affect beer quality, though gluten-free grains like quinoa yield lower alcohol and flavonoid content than barley-based beers.

Quinoa beer production involves processing, saccharification, fermentation, and packaging (26), with fermentation being the most critical stage. Yeast selection and fermentation conditions greatly influence beer quality (Table 1), affecting flavor and color, the main and secondary factors of total flavonoids content were fermentation temperature > feed water ratio > pH value. Lower fermentation temperatures increase flavonoid levels, enhancing antioxidant activity (29). Polyphenols possess antioxidant properties. During the fermentation process, a portion of polyphenols bind with proteins to form precipitates, resulting in a decrease in their content. β-Glucan undergoes degradation by β-glucanase during wort preparation and fermentation. Incomplete degradation can impact beer filtration and flavor (25). Bogdan et al. (30) observed that quinoa beer fermentation generates significant fatty and amino acids. In the fermentation process, phenolic compounds (conjugates with carbohydrates, fatty acids and proteins) existing in bound state will be transformed into free state under the action of chemical bond breaking of grain cell wall components and a variety of enzymes (β-glucosidase, decarboxylase, esterase, hydrolase and reductase). Free phenolic compounds have higher biological accessibility, and the released free aglycones can significantly improve antioxidant activity.

**Table 1:** Summary of quinoa-fermented products.

Category		Name	Technology	Characteristics	Changes in quinoa products before and after fermentation
Quinoa-wine	Quinoa beer	Non-alcoholic beer (26)	Pichia myanmarensis; 16 °C for 7 d and maturation at 4 °C for 14 d	Alcohol content < 0.5%vol.; pH 4.65; flavonoid content 18.51–30.85 mg L^−1^ Bitterness value (17.99–18.88 IBU) was lower than that of traditional beer; it presented a light golden to amber color. Esters and higher alcohols generated imparted fruity and roasted aromas, while ethyl propionate and heptanal endowed the quinoa beer with grape and herbal notes	FAN content decreased from 78.48 mg/L to 33.48 mg/L; total phenol content increased by approximately 30–50%; flavonoid content increased by about 50–86%; antioxidant activity (DPPH) improved by roughly 119–77%.
		Quinoa beer (27)	Saccharomyces pastorianus TUM 34/70; fermented at 16 °C for 2 d and statically fermented at 0 °C for 12 d	Alcohol content 5.91% vol.; pH 4.50; with nutty and strong quinoa aromas, grayish-yellow foam, slightly astringent taste accompanied by metallic and raw grass flavors, and rich in metal cations.	After fermentation, beer showed increased free amino acid nitrogen (FAN) and total soluble nitrogen (TSN), with FAN accounting for 64.8% of TSN. Total phenols increased from 13.85 to 16.53 mg GAE/g, while phytic acid decreased from 11.06 to 1.00 mg/g, tannins from 4.92 to 2.05 mg GAE/g, and saponins from 11.2 to 0.13 mg/g.
		Quinoa beer (28)	Dry powder yeast fermented at 20 °C for 8 d	Alcohol content 4.9% vol.; flavonoid content 230 mg L-1; pH value is 5.0; diacetyl content decreased to 0.10 mg/L; light yellow in color; with distinct vanilla flavor; beer foam is white and fine, with a foam retention of 260 s.	Flavonoid content decreased from 0.32 to 0.230 mg/mL
	Quinoa white wine	Quinoa liquid (32)	Medium-temperature *Daqu*; After 60 days of mixed steaming and continuous fermentation, it was stored in ceramic jars buried underground for 16 months	Mellow in the mouth; long aftertaste; strong-flavor liquor	
	Quinoa fruit wine	Quinoa ginseng wine (35)	Dry yeast; fermented at 32 °C for 9 d	Fermentation sugar content 25 Brix; Alcohol content 11.2% vol.; golden and bright color; mellow and harmonious taste, typical flavors of quinoa and ginseng fruit are prominent; Sensory score 89.11 points	After fermentation, the alcohol content reaches 45.09%, and there are 17 types of esters.
	Quinoa rice wine	Quinoa yellow wine (36)	*Saccharomyces cerevisiae* var. Ellipsoideus (yellow rice wine yeast strain), Sweet wine koji; First fermented at 30 °C for 5 d, then at 16 °C for 30 d	Alcohol content 10.3% vol.; pH 4.12; Non-sugar solid content 23.5 g/L; orange-yellow, clear and transparent, with unique oat fermentation aroma different from traditional rice yellow wine, mellow and refreshing taste; coconut aroma from α-amyl-γ-butyrolactone fermentation	After fermentation, the proportion of essential amino acids in total amino acids 33.64%; total polyphenol content: 295.01 μg/mL; 55 aroma components including phenylethanol (38.19%), isoamyl alcohol (17.45%), α-amyl-γ-butyrolactone (14.27%), etc.; esters and alcohols accounting for 27.39 and 27.39% respectively; alkaloid content before fermentation: 211.8 mg/kg, not directly detected in yellow rice wine after fermentation
		Quinoa yellow wine ()	*Saccharomyces cerevisiae*, *1,4-α-D-glucan glucanohydrolase*, *1,3-β-D-glucan glucanohydrolase*; liquefaction at 95 °C for 50 min, saccharification at 70 °C for 150 min, fermentation at 30 °C for 3 d	Alcohol content 9.6% vol.; Peptide content 4.95 g/L; low alcohol content; antioxidant activity	Total phenol and total flavonoid contents peaked on day 5 of fermentation at 163.75 μmol/100 g and 14.00 μmol/100 g·DW, respectively; polypeptide reached 4.95 g/L on day 4; DPPH free radical scavenging activity was the highest on day 3, ABTS free radical scavenging activity was the highest on day 4; FRAP iron reducing power remained at a high level from day 1 to 3, and decreased to 12.86 μmol/100 g on day 8
		Makgeolli (38)	*Lactobacilli, Saccharomyces cerevisiae*; fermented in a 25 °C incubator for 6 d	Alcohol content 10.3–10.7% vol.; pH 3.38–3.58; Brix value 6.40–8.70 Bx; Exhibits the cloudy appearance typical of traditional margaritas; nutty flavor; antioxidant function	After fermentation, DPPH free radical scavenging rate 81.01%; ABTS free radical scavenging rate 91.46%; iron ion chelating activity 47.09%; superoxide dismutase (SOD) activity 56.23%
Quinoa-fermented dairy products	Quinoa yogurt	Quinoa walnut yogurt (41)	Angel Yeast Strain 8 (*Streptococcus thermophilus*, *Lactobacillus delbrueckii* subsp. *bulgaricus*); fermentation at 42 °C for 6.1 h Sucrose addition amount 7.0%	Protein content 3.03 g/100 g; acidity 76 °T; using direct fermentation bacteria, containing a variety of fermentation bacteria and probiotics, with a clear aroma of quinoa and walnuts	
		Quinoa corn composite solidified yogurt (42)	*Streptococcus thermophilous* and *Lactobacillus bulgaricus*; fermentation at 45 °C for 8 h Sucrose addition amount 7.0%	Protein content 3.18 g/100 g; acidity 81.00 °T; pH 4.35; using Han Sheng gum and brown algae gum as stabilizers; a solidified yogurt made from green natural food	
		Quinoa-fermented yogurt (43)	Fermented bacterial powder (*Lactobacillus bulgaricus*, *Streptococcus thermophilus*, *Lactobacillus acidophilus*, *Lactobacillus plantarum*, *Lactobacillus casei*); fermented at 42 °C for 7.09 h	Protein content 5.2 g/100 g; acidity 76 °T; Yogurt viscosity (776.20 ± 8.50) MPa·s; hardness (0.13782 ± 0.0086) N; chewiness (5.2867 ± 0.035) mJ yogurt has a pure milk flavor and quinoa grain aroma, with rich nutrition	
		Quinoa yogurt (44)	*Streptococcus thermophilus* and *Lactobacillus bulgaricus*; fermentation at 43 °C for 3–4 h	pH 4.6–4.7	During the fermentation process, when the addition amount of quinoa flour was 1%, the hardness of yogurt gradually increased from 21,525 (Pa·sⁿ) to 40,105 (Pa·sⁿ); when the addition amount exceeded 1%, the hardness gradually decreased, and dropped to 16,943 (Pa·sⁿ) at 2%. The chewiness was the best when the addition amount of quinoa flour was 0.6%. However, when the addition amount was relatively high (e.g., 1.4 and 2%), the gel network became loose, the pores enlarged, and the structural stability decreased, resulting in poor chewiness.
		Quinoa ayran (45)	*Streptococcus salivarus* subsp. *thermophilus* and *Lactobacillus delbrueckii* subsp. *Bulgaricus*; fermentation at 4.6 (± 0.1) pH and 42 °C for 4–5 h, add 0.75% salt	Protein content 2.39%; dry matter 7.07%; pH 3.99; total phenol content100.01 mg GAE/L; antioxidant activity 19.25 μmol TE/L; texture showing non-Newtonian pseudoplastic flow	After fermentation, protein content increased by 16.02%; dry matter content increased by 6.16%; saturated fatty acid (SFA) content increased by 3.96%; monounsaturated fatty acid (MUFA) content decreased by 9.31%; total phenol content decreased by 55.62%; antioxidant activity increased by 20.31%; Consistency coefficient (K) increased from 0.09 mPa·sⁿ to 0.14 mPa·sⁿ
		Quinoa functional yogurt (47)	*Streptococcus thermophilus* and *Lactobacillus bulgaricus*; fermentation at 40–42 °C for 7 h	Protein content 3.02 g/100 g; acidity 82.00°T; fat content 1.85 g/100 g; finished product has moderate viscosity, lower fat content, and higher acidity compared to yogurt without added quinoa	
	Quinoa-fermented milk	‘Fruits and Vegetables—Kefir’ quinoa-fermented mixed milk (48)	*Lactobacillus kefiri* (ZKCC-378); 37.4 °C constant temperature cultivation for 12.4 h	Flavonoid content 0.853 mg mL^−1^; polyphenol content 10.361 mg mL^−1^; Sensory score:81.4 points; comprehensive score 63.0 points; high nutritional value	After fermentation, polyphenol content increased by 8.438 mg/mL, with a growth rate of 438.7%; flavonoid content increased by 0.515 mg/mL, with a growth rate of 152.4%; DPPH free radical scavenging rate increased from 52.3 to 85.6%; hydroxyl free radical scavenging rate increased from 32.5 to 61.1%; superoxide anion free radical scavenging rate increased from 41.2 to 65.1%; ferric reducing power increased from 0.325 to 0.711
		Sugar free quinoa-fermented milk (49)	*Lactobacillus plantarum* 117–1 and *Lactobacillus acidophilus* KLDS1.0901; fermented at 38 °C for 8 h	Fermented mixed grain milk with SOD enzyme activity 241.17 U/mL; pH 4.65; protein content 2.33 g/100 g; and *Lactobacillus* count of 5.85 × 109 CFU/mL; high antioxidant capacity	After fermentation, viscosity (2.52 Pa·s vs. 2.83 Pa·s) and water-holding capacity (59.46% vs. 64.28%) decreased; DPPH free radical scavenging rate increased from 76.81 to 86.23%; ABTS free radical scavenging rate increased from 52.96 to 71.32%; Fe^3^⁺ reducing capacity increased from 0.29 mmol/L to 0.48 mmol/L; hydroxyl free radical scavenging rate increased from 48.19 to 63.27%
		Quinoa-fermented milk (51)	*Streptococcus thermophilus*, *Lactobacillus acidophilus* La-5, and *Bifidobacterium animalis* spp. *lactis* BB-12; fermentation in batches at 42 °C three times until pH 4.6 After cooling, store at 4 °C for 28 d	*L. acidophilus La-5* and *Bifidobacterium animalis* have strong microbial activity	Lactic acid production increased with storage time, and the titratable acidity rose from 0.82% (Day 1) to 1.14%.
		Quinoa camel milk kishk (52)	*Streptococcus thermophilous*, *Lactobacillus bulgaricus*, *Lactobacillus plantarum* DSM 20205; milk incubated at 40 °C until pH4.5, then cooled down and stored at 5 °C; dough fermented at 37 °C for 24 h, then dried and stored for 8 weeks	Had a darker color (L value: 258–259), mild sourness (pH: 4.79–5.16), and a unique flavor; Protein content 22.10%; fat content 8.75%; rich in protein and hanve low fat quinoa camel milk kishk	After fermentation, protein content increased from 18.30 to 22.10%; carbohydrate content decreased by 39.43–46.37%; 19.72% of dietary fiber remained undegraded, which can maintain intestinal health; the viable count of *Lactobacillus plantarum* and other strains remained at 6.6–7.0 log CFU/g and remained active after 8 weeks of storage, ensuring intestinal health efficacy
		Quinoa kishk (53)	*Lactobacillus*; fermented at 37 °C for 24 h, then dried at 50 ± 2 °C for 48 h, and stored at room temperature for 3 months	Protein content 17.18–18.37%; fat content 3.00–5.99%; ash content 6.64–8.01%; fiber content 1.32–2.05%; pH 4.4; high nutritional content;50% quinoa substitution group (BQ) had the highest sensory score, with uniform texture and moderate sourness	Lysine content increased from 3.05 g/100 g protein to 6.17 g/100 g protein, with an increase of approximately 102%, improving amino acid balance; Fat content: increased from 2.82 to 5.99%; Carbohydrate content: decreased from 63.79 to 54.29%; Fiber content: increased from 1.18 to 2.05%; Calcium content: increased from 173.33 mg/100 g to 222.11 mg/100 g; Total phenol content: increased from 33.45 mg GAE/100 g to 111.64 mg GAE/100 g; IC₅₀ of DPPH free radical scavenging capacity: decreased from 290.27 mg/mL to 149.87 mg/mL
Other quinoa-fermented drinks	Quinoa-fermented tea	Red yeast quinoa-fermented tea (55, 56)	*Monascus purpureus* 3.4629; fermented at 31 °C for 10 d	pH 5.0; excellent antioxidant activity, the soup color is bright red, clear and transparent with a vivid hue; it has a rich quinoa aroma and ester fragrance, with a soft and pleasant taste	After fermentation, the final clearance rate increased from 44.25 to 52.12%
	Quinoa-fermented beverage	Quinoa-fermented beverage (60)	*Lactobacillus brevis* CGMCC 1.214, L*actococcus lactis* CGMCC 1.62; fermentation at 31 °C in fermentation tank for 22 h	GABA (0.681 ± 0.003) mg/mL; Viable count (9.176 ± 0.001) lg (CFU/mL); Moderate sour and sweet taste; unique fermentation flavor	During fermentation, glutamic acid decarboxylase from *Lactobacillus brevis* and *Lactococcus lactis* catalyzes glutamic acid in quinoa to generate GABA, with the content reaching (0.681 ± 0.003) mg/mL. GABA has physiological activities such as lowering blood pressure and improving sleep
		Black fungus quinoa compound fermented beverage (61)	Compound Lactic Acid Bacteria Powder (*Lactobacillus delbrueckii sub* sp. *bulgaricus*, *Streptococcus thermophilus*, *Bifidobacterium longum*, *Lactobacillus acidophilus*, *Lactobacillus plantarum*); fermented at 37 °C until pH < 3.5	Crude polysaccharides 864 mg/100 mL; protein 1.6%; L-lactic acid 8.7 g/L; pH < 3.5; uniform and fine texture, no precipitation or stratification, precipitation rate 0.51%; sweet and sour taste (sensory score 96), with natural flavors of black fungus and quinoa as well as lactic acid bacteria fermentation aroma	
		quinoa beverage fermented by a novel xylose-metabolizing *L. plantarum* strain (62)	*Lactiplantibacillus plantarum* P31891; Fermented at 30 °C for 48 h, then stored at 4 °C for 14 days	Lactic acid bacteria concentration up to 10^12^ CFU/mL; lactic acid content 0.5–1.0 g/100 mL; Increased the number of Lactobacilli in feces, decreasedthe number of pathogenic *Enterobacteriaceae*	pH 6.36–6.41 before fermentation, decreased to 4.03–4.04 after 48 h fermentation, and stabilized at 3.86–3.98 after 14 days of storage; pathogenic bacteria *Enterobacteriaceae* decreased from 2.9–3.1 log CFU/mL to <1 log CFU/mL, with a 90% reduction; lactic acid bacteria count increased from <1 log CFU/mL to 12.1–12.3 log CFU/mL, showing an increase of over 12 orders of magnitude
		Instant-mix plant-based fermented beverage (19)	*Lactobacillus plantarum* 299v Process 1: Inoculated with 0.1% bacteria after baking at 150 °C for 20 min, fermented at 37 °C for 9 h, and freeze-dried when pH < 4.6	pH 4.03; phytic acid 3.39 g/kg; lactic acid 90.9 g/kg; protein 139.7 g/kg Sour and sweet taste; reduced phytase content which can promote mineral absorption	After fermentation, phytic acid decreased by 61.8%, lactic acid increased by 321%, protein decreased by 2% after fermentation; molar ratio of phytic acid to iron (Phy: Fe) decreased from 14.10 ± 0.10 to 5.6 ± 0.13; molar ratio of phytic acid to zinc (Phy: Zn) decreased from 22 ± 0.00 to 9.3 ± 0.02, indicating promoted absorption of iron and zinc; viable count of lactic acid bacteria increased from 7.35 log_10_ CFU/g to 10.42 log_10_ CFU/g
			*Lactobacillus plantarum* 299v Process 2: First fermented at 37 °C for 6 h, then dried and baked at 150 °C, followed by fermentation at 37 °C for 3 h, and finally freeze-dried	pH 4.34; phytic acid 3.16 g/kg; lactic acid 124 g/kg; protein 143.3 g/kg Sour and sweet taste; reduced phytase content which can promote mineral absorption	After fermentation: phytic acid decreased by 64.4%, lactic acid increased by 474%, protein content remained unchanged; molar ratio of phytic acid to iron (Phy: Fe) decreased from 14.10 ± 0.10 to 5.3 ± 0.12; molar ratio of phytic acid to zinc (Phy: Zn) decreased from 22 ± 0.00 to 8.8 ± 0.01. Reduced phytic acid content alleviated its chelation with minerals such as iron and zinc, improving mineral bioavailability. Viable count of inoculated lactic acid bacteria increased from 7.35 log₁₀ CFU/g to 9.16 log₁₀ CFU/g
Quinoa-fermented food	Quinoa-fermented instant Congee	Low-GI coarse grain Congee (64)	Quinoa 35.00 g, oat 31.81 g, coix seed 16.75 g, buckwheat 16.44 g; cooked under normal pressure at a solid-to-liquid ratio of 1:12 (g/mL)	eGI value of Congee is 50.77,; Sensory score 87.03; which helps to stabilize the blood sugar concentration after meals; a low-GI food	
	Quinoa-fermented noodles	Quinoa-fermented pasta (62)	*Lactobacillus plantarum* T6B10 and *L. rossiae* T0A16; fermentation at 30 °C for 16 h	pH 4.02 ± 0.05; Protein content 12.3 ± 0.07 g/100 g; antioxidant activity 35%; cohesiveness 0.6%; dietary fiber 4.62 ± 0.10 g/100 g; lysine 34.0 ± 2.7 mg/kg; total phenols 4.06 ± 0.22 mmol/kg	After fermentation: water absorption rate reached 118.0 ± 4.5 g/100 g; hardness, fracturability and springiness all increased; protein increased by 20%; lysine increased by 7.39 times; total phenols and antioxidant activity increased by 83.7 and 150%, respectively; predicted glycemic index (pGI) decreased to 68.5 ± 0.5
		Quinoa pasta (68)	*Lactobacillus plantarum* CRL1964 and cRL2107; fermented at 30 °C for 24 h	pH 4.05–4.12; riboflavin (B_2_) 5.1 ± 0.4 μg/g; folic acid (B_9_) 1.6 ± 0.2 μg/g; magnesium content up to 264.3 ± 0.9 mg/L	Magnesium content increased by 23%, phytase degradation rate 49%; riboflavin and folic acid increased by approximately 132 and 3,100%, respectively
	Quinoa-fermented bread	Gluten-free bread (73)	*Lactobacillus sanfranciscensis* W2; fermented at 30 °C for 72 h	pH 4.53; total lactic acid bacteria 8.56 log₁₀ CFU/g; carbohydrates 60.0–74.7 g/100 g; firmness 0.098 ± 0.008c	Phytic acid content reduced by 30–50%; bread staling rate decreased by 20–30%; mineral bioavailability increased (e.g., dissolution rates of iron and zinc increased by 10–20%)
		Quinoa gluten-free bread (74)	*Weissella cibaria* MG1 and *Lactobacillus plantarum* FST1.7; anaerobically at 30 °C for 24 h	pH 3.9; α-amylase 0.17 IU/ g; protease 22.0 U/g; Protein content 14.61%; gumminess 951.81 (+106.43) gf	After fermentation: bread hardness decreased from 42.9 N to 24.0 N; porosity increased from 54.6 to 57.3%; cell volume enlarged by 61%, forming a looser structure
		Quinoa-fortified sorghum bread (75)	Microbial transglutaminase; Bake at 200 °C for 20–25 min after waking up with enzyme for 10 min	Protein 14.61%; Fat 10.38%; Ash 2.45%; Fiber 16.04%; Moisture 11.98%; Hardness 2,795.51 gf; Chewiness 971.56 gf; Gumminess 951.81 gf; Quinoa sorghum bread with good toughness but low hardness	Protein content increased by 1.25%; fat decreased by 26.59%; ash content increased by 4.70%; fiber significantly increased by 22.53%; moisture decreased by 21.18%; hardness significantly decreased by 20.32%; chewiness increased by 5.63%; gumminess increased by 65.74%; resilience significantly decreased by 28.93%; cohesiveness increased by 33.33% During fermentation, enzymes catalyze cross-linking reactions between glutamine and lysine, as well as between glutamine and water, forming a protein network, increasing protein content, enhancing protein hydration capacity, reducing bread moisture content to extend shelf life, and improving dietary fiber stability to facilitate its retention during baking
		Quinoa-fermented bread (76)	1.00% Saccharomyces; 5.48% butter, 75% humidity, first fermentation at 35 °C, second fermentation at 80% humidity and 38 °C for 30 min	Sensory score 95.9 points; Hardness 532.12 g; Soft taste and unique flavor	
		Quinoa bread (77)	0.5% *Saccharomyces cerevisiae*; 0.7% butter is fermented for 150 min in a 90% humid environment at a fermentation temperature of 32 °C	hardness 2,427 N; elasticity 714 mm; viscosity 366 mJ; moisture content 14%; protein content 1.6%; reducing sugar content 1.13%; sensory score 97 points	During fermentation, quinoa altered the network structure of gliadin-glutenin complexes, reducing the water-holding capacity of bread and thus affecting its water retention. Yeast consumed reducing sugars and fermented them into monosaccharides, resulting in a decrease in reducing sugar content
		Quinoa mixed grain bread (71)	1% *Saccharomyces cerevisiae*; Temperature 30 °C, 95% humidity, wake up for 66 min	Acidity 4.20°T ‘; specific volume 3.96 mL/g; hardness 13.83 N; elasticity 17.87 mm; chewiness 102.47 mJ	
	Mantou fermented with quinoa	Amorphophallus Mantou (72)	*Saccharomyces cerevisiae*; awakening at 75% humidity for 30 min at 34 °C	Low-GI Mantou rich in dietary fiber and protein	
		Chenopodium Mantou (78)	*Saccharomyces cerevisiae*; fermented at 75% humidity and 35 °C for 40 min	Hardness 14.36 N; elasticity 19.79 mm; chewiness 167.19 mJ; Specific volume 2.99 mL/g	
		Okra quinoa Mantou (79)	1.1 g *Saccharomyces cerevisiae*; 75% humidity, fermentation at 34 °C for 30 min, steaming time 15 min	Specific volume 2.35 mL/g; Hardness 12.41 N; Cohesiveness 0.71; Springiness 16.72 mm; Gumminess 9.09 N; Chewiness 152.35 mJ; Smooth surface, small and uniform internal pores, good springiness, moderate chewiness	
		Quinoa whole flour Mantou (81)	*Saccharomyces cerevisiae*; 38 °C, 80% humidity, wake up for 35 min, then steam for 20 min		
		Quinoa Mantou (82)	*Saccharomyces cerevisiae*; fermented at 30 °C for 20 min	Protein content 15%; moisture content 31.2%; volume 100 cm3	
		Quinoa Mantou (83)	*Saccharomyces cerevisiae* 4.8 g; 38 °C, humidity of 80%, awakening time of 15 min	Polyphenol content 165.41 mg GAE/100 g; ORAC value 2,624.4 μmol TE/100 g; Higher antioxidant activity of Mantou added with quinoa wheat flour	
		Quinoa whole flour Mantou (84)	*Saccharomyces cerevisiae*; temperature of 37 °C, fermentation time of 100 min, awakening time of 15 min	Hardness 9.76 N; chewiness 45.53 N; adhesive 6.66 N; sensory score 86.58 points	Water absorption rate increased from 55.75 to 59.75%
	Quinoa-fermented biscuits	Low-GI red yeast tea crackers (85)	*Monascus* sp. S2; wake up at room temperature for 30 min	Sensory score 92.7 points Crispy and delicious; light tea aroma and sweet taste; combination of low-GI and rich nutrition characteristics	
		Quinoa sourdough‐based biscuits (86)	*L. plantarum* CRL 1964, *Lc. mesenteroides* sub sp. *Mesenteroides* CRL 2131 fermentation at 30 °C for 24 h	pH 4.55–5.22; TPC 65.7–103.1 mg GAE/100 g; TAC 34.5–41.2 mg AAE/g	After fermentation, TPC in dough increased by 40–114%; free radical scavenging rate in biscuits increased by 10–15%
		Quinoa wheat bran high dietary fiber fermented biscuits (87)	*Saccharomyces cerevisiae*; first, ferment three times at 27 °C and 80% humidity, with fermentation times of 8 h, 8 h, and 1 h, respectively	Acidity 0.18%; moisture content 2.2%; dietary fiber content reaches 10.8 g/100 g; sensory score 85.8 points; high dietary fiber fermented biscuits	
	Other quinoa-fermented foods	Quinoa Tarhana (88, 89)	*Streptococcus thermophilus*, *Lactobacillus bulgaricus*, *Saccharomyces cerevisiae*; fermentation at 30 °C for 72 h	Total phenols 232.25 mg GAE/g; potassium 1,266.99 mg/100 g; TEAC 0.55 mmol TE/kg; Gluten free and nutrient rich	After fermentation, total phenol content and antioxidant capacity increased; phytic acid content increased linearly with the increase of quinoa flour amount (*p* < 0.05)
		Fermented spoonable vegan (90)	*Lactobacillus plantarum* Q823; fermentation at 30 °C for 6–8 h, stored at 6 °C for 28 days	pH 3.4–3.7; Viable count: ≥ 9 log cfu/g; Protein content 22%; potassium: 248.4 mg/100 g; fiber: 3.4 g/100 g; viscosity: 18.4–19.5 Pa·s; rich in fiber	After fermentation, lactic acid and acetic acid accumulate; TTA increases by 2–3 mL (0.1 M NaOH/10 g), and pH decreases from the initial 3.5–3.7 to 3.4–3.5, enhancing product acidity and preservability
		Gluten-free muffins (91)	*Lactobacillus plantarum* ATCC 8014; fermentation at 37 °C for 24 h	pH 4.2; glucose 22.00 mg/g; Lactic acid 8.50 mg/g f.w.; acetic acid 1.40 mg/g f.w.; folic acid: 648.39 μg/100 g f.w.; potassium 486.44 mg/100 g f.w.; flavonoids 1,551 mg Qe/100 g f.w.	After fermentation, glucose decreased from 42.034 mg/g f.w. to 22.00 mg/g f.w., while the content of organic acids such as lactic acid and acetic acid increased, which is related to the utilization of carbohydrates as carbon sources by fermenting bacteria; the reduction of carbohydrates provides a material basis for organic acid production, and the accumulation of organic acids lowers the pH of the system. The folate content in the dough increased significantly, which is associated with the ability of fermenting bacteria to synthesize B vitamins. The content of minerals such as potassium, magnesium, and calcium increased, with potassium increasing from 325.01 mg/100 g f.w. to 486.44 mg/100 g f.w., due to the acidic environment generated by fermentation reducing phytic acid content and promoting the release and absorption of minerals
Quinoa-fermented seasoning	Quinoa miso	Quinoa miso (92)	Aspergillus oryzae, Mucor sp., bacteria, Saccharomyces sp.; Fermented at 28–35 °C for 15–21 days for koji-making, followed by post-fermentation at 20 °C for over 40 days	VB₁ 0.28 mg/100 g; VB₂ 0.65 mg/100 g; Cellulose content 1.6–2.5%; Oil content 1.6–3.5%; Solid paste, fine and uniform; Flavor: mainly a harmony of umami, sweetness and saltiness, accompanied by ester aroma and mellow aroma produced by yeast fermentation	Total amino acids increased by 30–50% after fermentation; cellulose was partially degraded during fermentation, with simultaneous production of oligosaccharides (acting as bifidogenic factors) to regulate intestinal function. Oil content decreased slightly, while esters increased by 20–30% and the proportion of unsaturated fatty acids rose; phytase secreted by microorganisms decomposed phytic acid, increasing the solubility of minerals such as calcium and iron by 2–3 times and their bioavailability by 30–50%
		Quinoa miso (93)	Aspergillus oryzae AS3.951, *salt-tolerant lactic acid bacteria*, *Saccharomyces* sp. T; Fermented at 30 °C for 48 h for koji-making, followed by static fermentation for 4–5 months	Moisture ≤ 50%; Salt ≤ 10%; Amino acid nitrogen ≥ 0.16%	
	Quinoa sauce	Quinoa sauce (94)	Aspergillus oryzae; fermented at 28 °C with 90% humidity for 60 h, then further fermented at 35 °C for 35 d	pH 4.9; Cheng reddish brown or brownish yellow with rich aroma of soy sauce esters	
		Quinoa sauce (95)	mushroom mycelium; fermented at 25 °C for 14 days for koji-making, then further fermented at 28 °C for 2 month	The enzymes in the sauce are salt tolerant and contain a unique flavor of quinoa	
	Quinoa soy sauce	Quinoa soy sauce (96, 97)	Aspergillus oryzae, *Saccharomyces* spp.; fermentation enzymes using mechanical ventilation for constant temperature fermentation	Unique flavor; nutrient-rich	
Other quinoa-fermented products	Quinoa enzyme	Quinoa enzyme (99)	*Saccharomyces cerevisiae*; fermentation temperature 37 °C; fermentation time 48 h; inoculation amount 20%	Activity of quinoa enzyme lipase reached 67.76 U/mL; DPPH free radical scavenging rate reached 79.62%; good lipid-lowering and antioxidant abilities	
	Quinoa-fermented feed	Quinoa-fermented feed (100)	*Lactobacillus* sp. add 12 mg·kg^−1^; fermentation at 30 °C for 24.5 d	pH<4.0; Crude protein content 6.93%; Cellulose 33.88%; Hemicellulose 20.32%; Lignin 18.18%; easily digested and absorbed	After fermentation, the crude protein content of quinoa straw increased from 6.36% to (6.93 ± 0.05)%; the straw texture became “loose and soft,” with partial degradation of the fiber structure, further reduction in the resistance of lignin to enzymatic hydrolysis, and a decrease in pH to reduce methane emissions from the rumen of ruminants, facilitating their digestion and absorption
		Quinoa-fermented feed (101)	*Lactobacillus* sp. and *Saccharomyces* sp. Mixture, *Aspergillus niger*; Fermentation at 24.5 °C for 72–168 h	Increased crude protein and soluble sugar content in quinoa straw	After fermentation, crude fiber decreased to 20.03%; crude protein increased by 2.70%; crude fat increased by 0.24–0.31%; soluble sugar increased by approximately 3% on average

#### 3.1.2 Quinoa liquor

As a traditional Chinese distilled beverage, liquor undergoes solid-state fermentation through natural microbial inoculation with simultaneous saccharification and fermentation (31). Quinoa has proven to be an exceptional base material for liquor production. Yang et al.—developed China’s first strong-flavor quinoa liquor (“quinoa grain liquid”) using pure quinoa and medium-temperature wheat Daqu for saccharification and fermentation (32). This innovative spirit features a sweet, clean taste with a refreshing finish and remarkable persistence. While maintaining the characteristic robust, smooth profile of strong-flavor liquors, quinoa varieties develop unique flavor notes due to their specialized production methods. The choice of saccharifying and fermenting agents critically impacts the fermentation process. Consequently, brewers must carefully control koji fermentation parameters, duration, and temperature, as these factors directly determine the final product’s taste profile, aroma characteristics, and production yield (33).

#### 3.1.3 Quinoa fruit wine

Fermented fruit wine is typically made from fresh fruit through crushing, adjunct addition, fermentation, and aging. Many producers use mixed cultures of yeast and lactic acid bacteria to create more complex flavor profiles (34). The choice of yeast strain is particularly important as it directly affects the wine’s sugar and alcohol content. Kong et al. (35) developed quinoa ginseng wine by mixing quinoa and ginseng fruit (2:1 ratio), followed by liquefaction and saccharification. When the sugar concentration reached 25°Brix, they added 4% dry yeast and fermented the mixture at 32 °C for 9 days. The resulting wine had a golden hue, smooth taste, and unique blended flavor, with 11.2% alcohol content. Since quinoa contains high starch levels, amylase treatment is necessary for liquefaction and saccharification before fermentation. This process converts starch into fermentable sugars. While single-yeast fermentation yields higher sugar and alcohol concentrations, it often produces less complex aromas. In contrast, mixed yeast cultures degrade carbohydrates and proteins in raw materials through synergistic fermentation. Yeasts generate various esters via biosynthesis and the reaction between alcohols and free acids, thereby endowing the wine with characteristic flavors such as wine aroma, fruit aroma, and floral aroma. For instance, phenylethane exhibits a rose-like floral scent, while ethyl octanoate has an apricot aroma. These collectively result in a more balanced and richer flavor (35).

#### 3.1.4 Quinoa yellow wine

Yellow wine, a low-alcohol fermented beverage, preserves beneficial nutrients through fermentation. Liu (36) showed quinoa yellow wine contains higher essential amino acid levels than non-quinoa varieties, with superior nutritional and antioxidant properties compared to traditional millet yellow wine. Zhang et al. (37) developed quinoa wine using Qingli No. 2 through liquefaction (α-amylase 6 U/g at 95 °C for 50 min) and saccharification (glucoamylase 100 U/g at 70 °C for 150 min), followed by fermentation (4.0% yeast at 30C, 1:4 material-water ratio). Antioxidant activity peaked on day 3, with residual activity maintained thereafter. The initial fermentation phase (boiling chyme → mixing → fermentation → separation) is crucial for active compound formation, requiring stable temperatures. The liquefaction method’s slow fermentation with high yeast amounts yields lower alcohol content but better preserves quinoa proteins, it can also improve the fluidity of the wine body (37). Beyond these varieties, Jeon et al. (38) created Korean “makgeolli” using equal quinoa-rice proportions, demonstrating antibacterial, antioxidant and antitumor activities. These applications illustrate quinoa’s global potential as a wine fermentation substrate.

### 3.2 Quinoa-fermented dairy products

#### 3.2.1 Quinoa yogurt

Quinoa yogurt is produced through a standardized process involving quinoa preparation (soaking, boiling, blending), milk mixing, homogenization, sterilization, and fermentation with typical yogurt cultures (*S. thermophilus* and *L. bulgaricus*) (39, 40). Critical process parameters include fermentation time (6–8 h), inoculation amount (3.3–4%), and stabilizer addition (0.2% sodium alginate or Hansheng gum), which significantly influence product texture and flavor profiles (40, 41). Studies demonstrate that quinoa addition (optimal 0.6%) and sugar content (7%) are the most influential factors, affecting fermentation kinetics, acidity development, and final product viscosity (42–47). The recommended fermentation temperature ranges between 39 and 45 °C to ensure optimal microbial activity and product characteristics. Research indicates that quinoa incorporation modifies yogurt properties through multiple mechanisms: reducing pH, accelerating acidification, and enhancing texture by increasing the consistency coefficient (44–46). While quinoa substitution can shorten fermentation time by up to 25% compared to traditional formulations, excessive quinoa (>1%) or sugar concentrations may impair microbial growth and sensory attributes (45–47). Current optimization strategies focus on balancing quinoa content (0.6–1.0%), sugar levels (6–7%), and fermentation duration (6–8 h) to achieve ideal viscosity, flavor intensity, and nutritional enhancement while maintaining typical yogurt fermentation kinetics (43–47). During the fermentation process, proteases secreted by different fermenting bacteria decompose proteins in the raw materials into small-molecular peptides and amino acids. Small-molecular peptides possess a certain umami taste, while amino acids may participate in the Maillard reaction, thereby affecting the color and flavor of yogurt (41).

#### 3.2.2 Quinoa-fermented milk

Quinoa-fermented milk products are typically produced by lactic acid bacteria fermentation using quinoa pulp and reconstituted milk (48). Studies demonstrate that these products exhibit enhanced functional properties compared to conventional fermented milks. Chen et al. developed a “fruit and vegetable-kefir” quinoa-fermented milk with significantly higher polyphenol and flavonoid contents, along with superior antioxidant capacity (hydroxyl radical, DPPH radical, and superoxide anion radical scavenging rates) and iron reduction ability (48). Optimization studies by Zhang et al. identified fermentation temperature as the most critical factor affecting superoxide dismutase (SOD) activity in sugar-free quinoa-fermented milk (prepared with 30% quinoa pulp, 5% xylitol, and a 2:1 *L. plantarum* to *L. acidophilus* ratio at 38 °C for 8 h) (49). The incorporation of quinoa pulp has been shown to modify milk’s physicochemical properties while boosting antioxidant activity (50, 51). Beyond traditional lactic acid bacteria, innovative applications utilize probiotic strains (*Lactobacillus* and *Bifidobacterium*) to develop functional variants like quinoa-enriched MILK KISHK, with demonstrated improvements in protein content (52) and shelf life (53). The fermentation process naturally reduces water activity (AW) and pH, inhibiting spoilage microorganisms while promoting beneficial flora that enhance intestinal health and mineral absorption. Nutrients such as polysaccharides, polyphenols, saponins, and bioactive peptides in quinoa, after being fermented by intestinal flora, can selectively promote the proliferation of beneficial bacteria like *Bifidobacterium* and *Lactobacillus*. Meanwhile, the content of dietary fiber in fermented quinoa remains undegraded, which is beneficial for maintaining intestinal health. These findings underscore the importance of starter culture selection in optimizing both product quality and health-promoting properties.

In summary, quinoa-based fermented dairy products, including yogurt and fermented milk, demonstrate enhanced nutritional and functional properties through optimized fermentation processes. These products represent promising functional food innovations that combine traditional dairy fermentation with quinoa’s nutritional advantages, though careful control of formulation parameters remains crucial for optimal quality and sensory acceptance.

### 3.3 Other quinoa-fermented drinks

#### 3.3.1 Quinoa-fermented tea

Quinoa fermented tea demonstrates superior nutritional and bioactive properties compared to traditional baked or stir-fried quinoa tea. Quinoa leaves contain valuable components including 3.3% ash, 1.9% fiber, 0.4% nitrate, and 0.29% vitamin E, along with abundant proteins and lipids, making them ideal for tea production (54). Quinoa tea can be processed into two types: black tea and green tea. The manufacturing process of quinoa green tea is as follows: spreading and air-drying → de-enzyming → drying. Among these steps, de-enzyming is critical. Enzymes are inactivated at a high temperature of 230–250 °C to retain the green color and nutrients of the tea, which is finally dried at 100–140 °C. The manufacturing process of quinoa black tea involves withering → rolling → fermentation → drying. The fermentation is carried out under conditions of different temperatures (38–50 °C) and humidities (20–80%) to activate enzyme activity and promote the transformation of substances in the tea, and the final product is obtained by drying at 80–100 °C. The characteristics of quinoa tea are essentially the result of the combined effects of “physical treatment—enzyme activity regulation—substance transformation” during the tea-making process. Green tea is centered on “inhibiting enzyme activity and retaining natural components,” while black tea is keyed on “guiding enzymatic reactions and reorganizing functional substances.” The rich chemical changes during these fermentation processes endow quinoa tea with unique nutritional properties and flavors.

Fermentation enhances flavor profiles, with He et al. (55) identifying 17 distinct flavor compounds in fermented quinoa compared to only 16 in unfermented samples. A notable preparation method developed by Ling et al. (56) involves combining quinoa with *Monascus*, incubating at 31 °C for 10 days to produce red yeast rice-quinoa fermented powder, which when mixed with cooked quinoa (1:25 ratio) yields tea with significantly higher antioxidant activity than conventional baked quinoa tea. The fermentation process substantially increases soluble phenolic compounds including vanillic acid, protocatechuic acid, and rutin (56), while simultaneously reducing bitterness and improving overall quality (57). However, challenges include potential flavor loss and susceptibility to mildew during fermentation, necessitating strict control of humidity and fermentation duration to maintain product quality and stability. These findings highlight the importance of optimized fermentation parameters for developing high-quality quinoa tea products with enhanced nutritional and sensory characteristics.

#### 3.3.2 Quinoa-fermented non-alcoholic beverages

Quinoa-fermented non-alcoholic beverages, produced through microbial fermentation, offer notable health benefits including gastrointestinal regulation and improved digestion, Glutamate decarboxylase from fermentative bacteria catalyzes the conversion of glutamic acid in quinoa to GABA, and GABA possesses physiological activities such as reducing blood pressure and improving sleep. The standardized production process involves quinoa processing, microbial inoculation, fermentation, broth preparation, and flavor blending (58–60). Microbial selection critically determines beverage characteristics, as demonstrated by Yang et al. (60) using 4% lactic acid bacteria (34 °C, 24 h) to achieve balanced sour–sweet flavors, while Liu et al. (61) combined Auricularia auricula with quinoa and 5 g/L lactic acid bacteria (37 °C) for natural flavor development. Novel strains like P 31891 significantly enhance fermentation efficiency (62), and *L. plantarum* 299 V (37 °C, 9 h) reduces phytase content, potentially improving mineral absorption (19). These microbial metabolic processes transform carbohydrates into organic acids, the decrease in pH activates endogenous phytase in quinoa, promoting phytate degradation and thereby enhancing the absorption of nutrients. Both enzymatic reactions and microbial activities can significantly improve antioxidant activity while altering flavor characteristics (63). Optimal strain selection thus enables tailored development of beverages with specific functional and sensory properties.

Overall, quinoa-fermented tea and non-alcoholic beverages represent innovative functional products where microbial fermentation significantly enhances both nutritional value and sensory quality. Future research should focus on strain-specific metabolic pathways to further optimize the fermentation-mediated bioconversion of quinoa’s unique phytochemicals, particularly exploring synergistic effects between traditional tea-processing techniques and modern microbial fermentation technologies for developing next-generation functional beverages. This approach could bridge traditional consumption patterns with contemporary health demands while addressing current challenges in flavor consistency and process standardization.

## 4 Quinoa-fermented foods

Quinoa has emerged as a valuable raw material for diverse food products due to its rich nutritional profile and suitability for low-GI formulations. However, its high dietary fiber content often results in technical challenges including low specific volume, dry texture, and coarse mouthfeel, which negatively impact sensory quality. Fermentation presents an effective solution to these limitations, simultaneously enhancing palatability while boosting nutritional value and bioactive properties. During the fermentation of quinoa, the content of free phenolic acids and flavonoids will significantly increase, the content of total polyphenols will increase by 46.56%, the content of total flavonoids will increase by 57.28%, and the content of bound phenols and polymers will decrease, so the antioxidant capacity of quinoa after fermentation will be significantly improved (*p* < 0.05) (60). The demonstrated improvements in both organoleptic characteristics and functional benefits suggest quinoa-fermented foods hold significant potential for future food development, warranting expanded research efforts in this promising field.

### 4.1 Quinoa-fermented instant porridge

While quinoa porridge serves as a nutritious low-GI food option, its inherent hardness presents textural challenges that conventional processing methods fail to adequately address while remaining time-consuming. Fermented instant porridge technology emerges as an effective solution, significantly improving both convenience and palatability (64). Under the slow action of quinoa’s own endogenous enzymes, starch is decomposed into maltose, oligosaccharides, etc., which slows down the postprandial blood glucose rise. The water-soluble dietary fibers abundant in quinoa absorb water and swell to form a viscous gel layer, which wraps starch granules and hinders the contact between amylase and starch, thereby delaying the digestion and absorption of carbohydrates. Advanced formulations utilizing specialized starters like selenium- and chromium-enriched *S. cerevisiae* demonstrate particular promise, offering enhanced nutritional balance and metabolic benefits that make them especially suitable for individuals managing hypertension, hyperlipidemia, and diabetes. These technological advancements position fermented quinoa porridge as a practical and health-promoting food alternative.

### 4.2 Quinoa-fermented noodles

Quinoa’s nutritional profile, characterized by high dietary fiber and low GI value, makes it particularly suitable for developing healthy noodle products (65). Fermentation significantly enhances the structural and nutritional qualities of quinoa-based pasta, with studies demonstrating improved elasticity and reduced phytase content through lactic acid bacteria processing (66, 67). Notably, specific *L. plantarum* strains (CRL1964 and CRL2107) enable effective vitamin B2 and B9 biofortification, offering potential solutions for micronutrient deficiencies as evidenced in animal studies (68, 69). Successful quinoa noodle production requires strategic selection of both raw materials and fermentation strains to optimize both nutritional value and consumer acceptability, with current research primarily focused on lactic acid bacteria applications for quality enhancement.

### 4.3 Quinoa-fermented bread

Quinoa-enriched bread has emerged as a nutritionally superior alternative to conventional wheat bread, offering enhanced aroma, texture, and functional properties (70). The fermentation process and final product quality are influenced by multiple factors including quinoa flour ratio (optimal 12%), butter content (0.7–5.48%), and specialized starter cultures (71, 72). Recent innovations include gluten-free formulations using *L. plantarum* CRL 778 for celiac patients (73), improved textural properties through lactic acid bacteria fermentation (reduced hardness, enhanced phytase activity) (74), and superior crumb structure via exopolysaccharide-producing *Weissella* MG1 (75, 76). Advanced techniques like microbial transglutaminase fermentation significantly increase protein/fiber content while improving dough rheology (77). Optimal production parameters involve two-stage fermentation systems [35–38 °C, 75–90% Relative humidity (RH)] with precisely controlled ingredient ratios (71, 72), where temperature primarily affects cohesiveness/elasticity and butter concentration determines hardness. During the fermentation process, organic acids in quinoa can inhibit starch retrogradation, thereby reducing the staling rate of quinoa bread. Additionally, organic acids can ferment in sucrose to synthesize dextran, which acts as a hydrocolloid to improve the water-holding capacity of dough and increase the porosity of bread simultaneously. These technological advancements, combined with strict process control to prevent structural defects, position quinoa bread as a promising functional bakery product with enhanced nutritional value, sensory quality, and extended shelf stability.

### 4.4 Quinoa-fermented steamed buns

The growing demand for nutritious bakery options has spurred the development of low-GI quinoa steamed buns, with research demonstrating significant improvements in formulation and processing techniques. Optimal proofing conditions (temperature 28–38 °C, humidity 75–80%) critically influence texture and fluffiness (78), while specialized yeast strains enhance water-binding capacity and viscoelasticity (79). Innovative formulations include konjac-quinoa-protein blends (34 °C, 75% RH, 30 min) for increased fiber/protein content (80) and okra-quinoa combinations (28 °C, 80% RH, 20 min) for nutritional enhancement (81). Studies establish ideal parameters as 10–20% quinoa flour with 0.75–1% yeast and 15–35 min proofing times (82–84), where black quinoa (20% incorporation) shows superior antioxidant activity (83). Maintaining quinoa content below 20% preserves gluten network integrity, while standardized yeast concentrations (1%) ensure optimal texture. These advancements enable production of functional steamed buns with improved nutritional profiles, sensory qualities, and reduced proofing times through controlled temperature/humidity conditions.

### 4.5 Quinoa-fermented cookies

Traditional fermented biscuits often suffer from nutritional imbalances, featuring excessive carbohydrates, sugars and fats alongside insufficient vitamins and quality proteins. Quinoa incorporation addresses these limitations while enhancing functional properties. Zhou et al. developed *Monascus*-fermented quinoa biscuits using tea byproduct culture media, achieving desirable crispness and distinctive tea aroma suitable for low-GI diets (85). Sandez Penidez et al. created lactic acid bacteria-fermented variants with elevated antioxidant activity, potentially replacing synthetic antioxidants (86). Qiu et al. pioneered high-fiber quinoa bran biscuits (10.8% dietary fiber) through triple fermentation of wheat flour, quinoa flour and bran (87). These advancements demonstrate quinoa-fermented biscuits’ superior nutritional profiles, improved sensory characteristics, and functional benefits including enhanced antioxidant capacity, delayed lipid/protein oxidation, and extended shelf stability, positioning them as healthier alternatives in the baked goods sector (86).

### 4.6 Other quinoa-fermented foods

Quinoa has been successfully adapted to diverse fermented food applications beyond traditional products. In Turkish tarhana production, partial substitution of wheat flour with quinoa enhances nutritional and rheological properties while preserving sensory acceptability (88, 89). Fermentation effectively transforms quinoa’s characteristic earthy flavor into pleasant fermented notes, as evidenced in Väkeväinen et al.’s development of probiotic-enriched vegan quinoa snacks (90). Additionally, Chiş et al. demonstrated quinoa’s potential in specialized dietary products through *L. plantarum* ATCC 8014-fermented quinoa flour muffins, which exhibit reduced carbohydrates alongside elevated organic and folic acid content (91). These applications highlight quinoa’s versatility in meeting contemporary food innovation demands while improving nutritional profiles.

Quinoa fermentation technology effectively addresses the inherent textural and sensory limitations of quinoa-based foods while significantly enhancing their nutritional and functional properties. Future research should focus on elucidating the molecular mechanisms underlying fermentation-induced nutrient transformations, particularly the interplay between microbial consortia and quinoa’s unique phytochemical matrix, to develop next-generation functional foods targeting specific metabolic disorders. This approach will facilitate the transition from empirically optimized processes to rationally designed fermentation systems that maximize both nutritional and commercial potential.

## 5 Quinoa-fermented condiments

### 5.1 Quinoa miso

Quinoa has been successfully adapted for miso production (known as “doenjang” in China), a traditional Japanese fermented condiment valued for its nutritional benefits, immune-modulating properties, and potential cancer-preventive effects (92). Liu et al. developed an optimized production protocol involving: (1) quinoa cooking and Aspergillus oryzae inoculation (30 °C koji production), followed by (2) mixing with cooked soybeans (2:1 ratio) and (3) anaerobic fermentation with yeast/lactic acid bacteria (28–32 °C, 4–5 months) to yield a glossy, aromatic reddish-brown product (93). Critical parameters include strict temperature control (28–32 °C) and anaerobic maintenance to prevent undesirable yeast byproducts (92). This quinoa incorporation not only enhances miso’s nutritional profile but also expands product diversity, demonstrating significant potential for fermented food industry innovation.

### 5.2 Quinoa sauce

Quinoa sauce quality and flavor development depend critically on koji preparation, fermentation techniques, and aging processes, with the aging stage being particularly vital for protein denaturation and enzymatic hydrolysis (94). Nakamura et al. developed an innovative mushroom mycelium-derived fermentation enzyme (25 °C, 14-day cultivation) that significantly enhances quinoa’s flavor profile (95). Comparative studies by Dong et al. demonstrated extrusion puffing as the optimal aging method, producing sauce with superior moisture content (15–18%), total acidity (1.2–1.5%), amino acid nitrogen (0.8–1.2 g/100 g), and reducing sugars (3.5–4.0%) while maintaining ideal pH (4.5–5.0) and salt levels (12–14%) (94). Fermentation reduces the hardness of quinoa products by 25–40% by degrading cellulose and pectin. The organic acids (lactic acid, acetic acid) produced by fermentation increase the acidity value. At the same time, the alcohols and esters produced by fermentation impart a floral and fruity aroma, which can mask the grassy taste of the original quinoa. These technological advances not only improve nutritional value and flavor complexity but also create new opportunities in the growing multigrain condiment market, particularly for health-conscious consumers seeking innovative fermented products.

### 5.3 Quinoa soy sauce

Quinoa-enriched soy sauce merges traditional fermentation techniques with modern nutritional enhancement, employing distinct regional methods: high-salt dilute-state fermentation (Japan/Korea) and combined high-salt/low-salt approaches (China) (96). The production process combines quinoa with steamed soybeans using *A. oryzae* and specialized yeast strains, yielding products with improved nutritional profiles and unique flavor characteristics. Technological advancements include temperature-controlled mechanical ventilation for optimized koji production and low-temperature fermentation to enhance *A. oryzae* activity (97). The incorporation of salt-tolerant *Torulopsis* yeast and *Rhodotorula* species further improves lipid metabolism and aroma development (97), demonstrating quinoa’s potential to expand both functional and sensory properties in traditional soy sauce fermentation while maintaining authentic production methods.

Quinoa-fermented condiments, including miso, sauce, and soy sauce, demonstrate significant potential in combining traditional fermentation techniques with modern nutritional enhancement. The incorporation of quinoa not only diversifies product offerings but also addresses growing consumer demand for innovative, health-promoting fermented foods while maintaining traditional production authenticity. Future research should focus on standardizing processes and exploring additional functional benefits of quinoa in fermented condiments.

## 6 Other quinoa-fermented products

### 6.1 Quinoa enzymes

Microbial fermentation employing lactic acid bacteria, molds, and yeasts drives essential biochemical conversions that produce bioactive enzymes and metabolites (98). This process significantly enhances product quality by modifying flavor profiles, intensifying coloration, reducing irritant compounds, and generating novel bioactive substances including flavonoids and organic acids. In quinoa-specific applications, Tian et al. established optimal fermentation conditions (37 °C, 48-h duration, 20% yeast inoculum) that maximize these beneficial transformations, demonstrating the potential for precisely controlled microbial processes in developing functional quinoa-based food products with enhanced nutritional and sensory properties (99).

### 6.2 Quinoa-fermented feed

Quinoa straw offers superior nutritional characteristics and digestibility compared to corn straw due to its lower lignin content (100). Fermentation processing enhances these properties, improving texture and palatability while increasing nutritional value. Lv et al. determined the key fermentation parameters in order of significance: lactic acid bacteria concentration > moisture content > duration, with optimal conditions (12 mg/kg inoculum, 60% moisture, 24.5 days) yielding 6.93% crude protein (100). Comparative studies by Yu et al. demonstrated fermentation’s nutritional benefits, including 2.70% crude protein increase, approximately 3% higher soluble sugars, and enhanced cellulose degradation versus unfermented feed (101). These improvements position fermented quinoa straw as a viable alternative feed source with enhanced digestibility and nutritional profile.

## 7 Key technologies and applications of quinoa fermentation

### 7.1 Impact of the sensory properties of fermented quinoa products on consumer preferences

The application of fermented quinoa products has garnered increasing attention; however, the relationship between their sensory characteristics and consumer preferences exhibits complexity, influenced not only by the inherent properties of quinoa but also by processing techniques, incorporation ratios, and regional cultural differences (102). Studies indicate that the texture and flavor of fermented quinoa products are critical determinants of consumer acceptance, with negative sensory attributes such as off-flavors and grittiness significantly reducing preference (103). Meanwhile, the use of flavoring agents, such as adding raspberry syrup to fermented quinoa beverages, can markedly enhance product acceptability (104).

Furthermore, the incorporation ratio of quinoa exhibits a threshold effect on consumer preference, with low-to-moderate levels (5–30%) generally being well-accepted, whereas exceeding a certain limit leads to decreased acceptability (102). Cross-cultural studies have also demonstrated significant regional variations in preferences for quinoa-based products, underscoring the importance of market-specific optimization (105). Notably, while quinoa can enhance the nutritional profile of food products, this improvement may sometimes come at the expense of sensory quality, particularly in low-fat or gluten-free formulations (106).

In summary, during the development of fermented quinoa products, maintaining sensory quality alongside nutritional enhancement is essential, and formulations should be tailored to the specific demands of target markets. Future research should focus more on balancing the health benefits of quinoa with sensory acceptability, particularly in product development for children, aiming to foster long-term consumption habits (102, 106).

### 7.2 Impact of fermented quinoa products on human health

Fermented quinoa products demonstrate significant potential in modulating gut microbiota, enhancing the biotransformation of bioactive compounds, and improving human health. Studies indicate that quinoa-derived polysaccharides and dietary fibers act as prebiotics, reducing intestinal pH to promote the proliferation of beneficial bacteria such as *Bifidobacterium* while inhibiting pathogens like *Escherichia coli* (6). Additionally, quinoa flavonoids can modulate gut microbiota composition by increasing the abundance of Firmicutes while reducing Bacteroidetes, thereby improving microbial balance (22). The short-chain fatty acids (SCFAs) produced during fermentation not only help maintain intestinal barrier integrity but also regulate immune and inflammatory responses by activating G protein-coupled receptors (GPCRs) (107, 108).

Regarding the biotransformation of bioactive compounds, microbial fermentation significantly enhances the total phenolic content and bioavailability in quinoa, particularly elevating the levels of flavonoids such as quercetin and kaempferol, which strengthens antioxidant activity (23, 109). Fermentation also degrades toxic alkaloids in quinoa saponins, generating low-molecular-weight sapogenins, while hydrolyzing polysaccharides into readily fermentable oligosaccharides, further promoting SCFA production (22, 110). These metabolites exert beneficial effects on human health, including ameliorating intestinal disorders, mitigating metabolic diseases, and providing systemic protection. For instance, SCFAs have been shown to alleviate intestinal damage in models of inflammatory bowel disease (IBD) and colorectal cancer, while modulating the gut-brain axis to improve symptoms of irritable bowel syndrome (IBS) (22, 62, 107).

Despite challenges such as dose-dependent potential toxicity and individual variability, the therapeutic effects of fermented quinoa products on metabolic disorders—including obesity, diabetes, and non-alcoholic fatty liver disease (NAFLD)—have been preliminarily validated in animal studies (22, 111). Future research should focus on elucidating the interaction mechanisms between fermentation strains and quinoa components, optimizing the synbiotic effects of prebiotics and probiotics, and conducting long-term human clinical trials to validate the health benefits of fermented quinoa products (22, 62).

### 7.3 Effects of bacterial strains during quinoa fermentation

Specific microbial strains play pivotal roles in quinoa biotransformation through multiple mechanisms that promote growth, enhance stress resistance, suppress diseases, and regulate metabolic processes. Plant growth-promoting rhizobacteria (PGPR) such as *Bacillus licheniformis* QA1 and *Enterobacter asburiae* QF11 secrete organic acids to solubilize insoluble phosphates in soil, improving phosphorus availability and thereby stimulating quinoa root development and biomass accumulation (112). Additionally, nitrogen-fixing strains can fix atmospheric nitrogen, providing supplemental nitrogen nutrition that significantly enhances plant height, panicle weight, and grain yield (113, 114).

The synthesis and signaling of phytohormones constitute another critical mechanism influencing quinoa growth. For instance, *Pseudomonas* sp. M30-35 produces indole-3-acetic acid (IAA), directly stimulating cell division and root elongation to improve nutrient uptake efficiency (115). Seed coating with *Trichoderma harzianum* TE-7/TE-126 activates phytohormone signaling pathways, increasing shoot dry weight and yield by 2- to 3-fold (116).

In terms of stress resilience enhancement, specific strains significantly improve quinoa’s tolerance to environmental challenges. Under salt stress, *Pseudomonas* sp. M30-35 maintains photosynthetic efficiency by increasing chlorophyll a/b content while promoting root activity and saponin accumulation to counteract phosphorus deficiency (115). Furthermore, the mineral-solubilizing bacterium *Pontibacter lucknowensis* Cq-48 enhances water-use efficiency by improving transpiration rates and leaf area (114).

Biocontrol and disease suppression represent another key function. Trichoderma spp. exhibit mycoparasitism by coiling around and lysing pathogenic hyphae, reducing downy mildew incidence, while their volatile organic compounds (VOCs) demonstrate significant inhibitory effects against Botrytis cinerea (117, 118). Endophytic bacteria secrete antimicrobial metabolites to mitigate bacterial leaf spot severity (119). Additionally, these microbes induce systemic resistance by upregulating defense-related genes, strengthening quinoa’s tolerance to pathogens (119).

Finally, fermentation enhancement mechanisms reveal that *lactic acid bacteria* (LAB) such as *Lactobacillus fermentum* and *Lacticaseibacillus rhamnosus* can significantly elevate α-glucosidase and α-amylase inhibitory activities during quinoa seed fermentation, thereby improving anti-hyperglycemic functionality (120).

In summary, specific microbial strains exert multifaceted interactions to drive quinoa biotransformation. Future research should focus on optimizing their application strategies to achieve sustainable quinoa production (113, 115, 116).

### 7.4 Industrial and therapeutic applications of quinoa fermented products

Quinoa, as a highly nutritious pseudocereal, demonstrates remarkable potential in industrial applications and therapeutic uses through its fermented products. Rich in bioactive components including proteins, polyphenols, saponins, and dietary fibers, quinoa undergoes microbial biotransformation during fermentation, thereby enhancing its functional properties (121).

In industrial applications, fermented quinoa beverages not only exhibit improved foam stability but also serve as wine-clarifying agents to enhance product texture and sensory characteristics (18). Furthermore, fermented quinoa byproducts can be utilized to produce bio-preservatives, emulsifiers, or food carriers, suitable for baked goods and plant-based milk alternatives, offering new options for lactose-intolerant individuals (121, 122).

Regarding therapeutic applications, fermentation significantly enhances the antioxidant, antihypertensive, and antidiabetic activities of quinoa extracts (121). Studies indicate that probiotic fermentation of quinoa water-soluble extracts increases polyphenol and peptide content, effectively scavenging free radicals while inhibiting α-glucosidase and pancreatic lipase, suggesting potential interventions for metabolic disorders (121). Additionally, saponins and polysaccharides in fermented quinoa products exhibit anti-inflammatory and antimicrobial properties, making them promising candidates for neuroprotective agents or immune enhancers (121, 123).

Future research should prioritize the following key areas: First, novel bioactive compounds-particularly phytochemicals from diverse quinoa genotypes or regional varieties-need to be isolated and characterized, with emphasis on their transformation mechanisms during fermentation (121). Second, green and sustainable processes should be optimized to recycle quinoa byproducts, reducing industrial costs and environmental footprints (121). Third, clinical validation and safety assessments are critical, as current studies predominantly rely on *in vitro* or animal models; human clinical trials are warranted to confirm the safety and therapeutic efficacy of fermented products (121). Lastly, expanding applications in emerging fields-such as nanocarriers for drug delivery or edible films-and integrating biotechnology to enhance stress-resistant quinoa varieties should be explored (121, 122).

**Figure 2:**
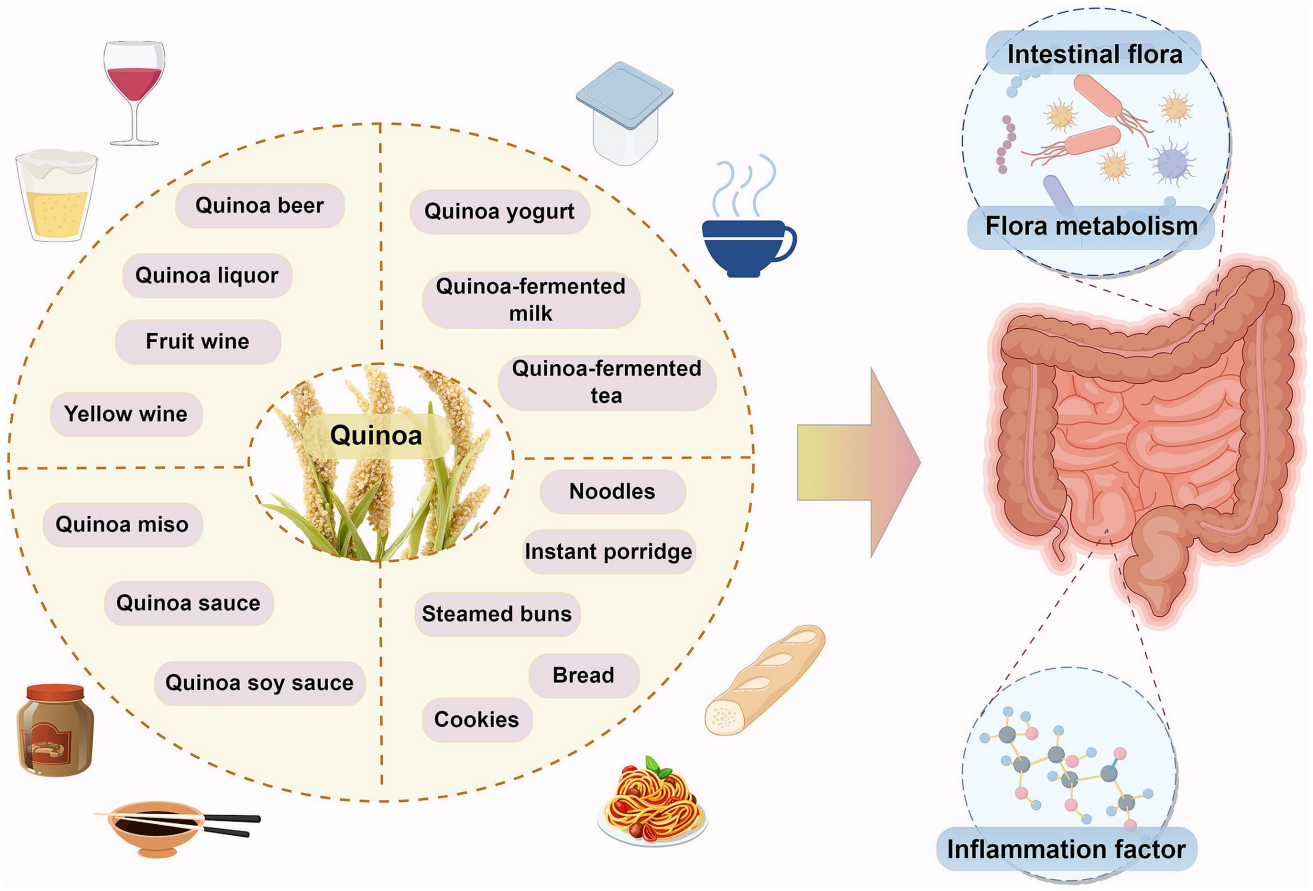
A summary of the findings discussed in this review. This figure was drawn by Figdraw.com and some images by OpenClipArt-vectors and Tshirtshophoplix via Pixabay.com.

In summary, fermented quinoa products exhibit broad prospects in industrial and therapeutic applications. However, future research must address bottlenecks including varietal diversity, process optimization, and clinical translation to facilitate the transition from laboratory to market (121, 122) (Figure 2).

## 8 Future perspectives and innovations

Current research has primarily focused on grain wines and dairy products, with fermented teas and condiments receiving comparatively less attention. To advance this field, future efforts should focus on three key directions:

(1) Process optimization and standardization

Given the substantial variation in fermentation parameters, systematic optimization is critical to enhance efficiency and reproducibility. Reducing processing time by 30–50% through strain selection, enzyme supplementation, or dynamic fermentation control could significantly improve economic viability, which is currently hampered by a 20–40% cost increase due to prolonged fermentation.

(2) Sensory and nutritional enhancement

Further research should elucidate the microbial mechanisms governing flavor development and texture modulation to align with regional dietary preferences. Targeted fermentation using functional strains may concurrently improve palatability, gut health benefits, and nutrient bioavailability.

(3) Market-driven product diversification

Expanding beyond conventional formats, localized adaptations-such as savory condiments for Asian markets or high-protein fermented snacks for Western consumers-could boost market penetration. Integrating multi-omics approaches will accelerate strain screening and process design, facilitating scalable production while preserving nutritional advantages.

## 9 Conclusion

**Figure 3:**
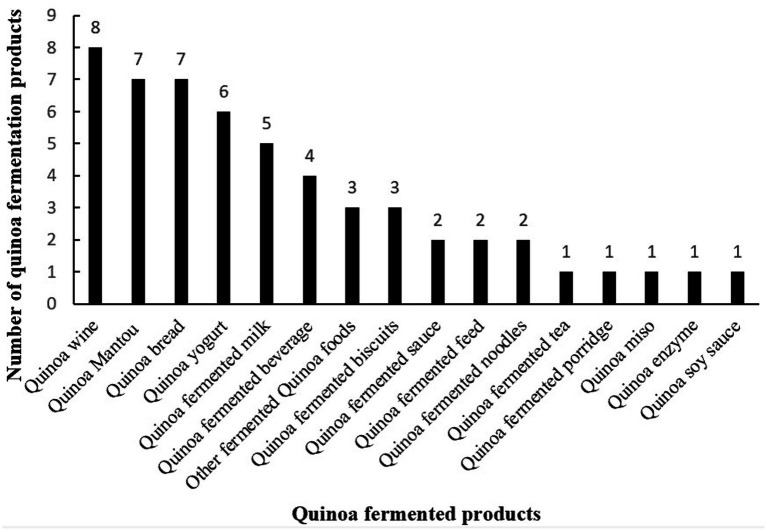
Types and quantities of quinoa fermented products.

This study systematically cataloged 54 quinoa fermented products developed worldwide (Figure 3). Analysis of these products revealed distinct fermentation protocols: alcoholic products employ low-temperature fermentation (<30 °C) with specific microbial consortia (yeast for beer/fruit wine, Daqu for Baijiu, combined enzymes/yeast for yellow wine), while dairy products utilize thermophilic bacteria (*S. thermophilus*/*L. bulgaricus* at 42 °C for 6–8 h for yogurt; *Lactobacillus*/*L. plantarum* at 37–42 °C for fermented milk). Bakery products demonstrate optimal fermentation at 30–38 °C with 75–80% RH for 15–40 min. This comprehensive process inventory establishes critical reference parameters for: (1) nutrient preservation through optimized temperature control, (2) standardization of microbial starter cultures, and (3) industrialization potential assessment. The findings particularly highlight lactic acid bacteria’s dual utility in both human food and animal feed fermentation, suggesting cross-industry applications. These documented processes provide a foundational framework for scaling production while maintaining product quality, addressing current challenges in quinoa fermentation standardization and commercial viability. Future research should focus on mechanization adaptations of these optimized protocols to facilitate industrial adoption.
